# Pathophysiology of high fat diet induced obesity: impact of probiotic banana juice on obesity associated complications and hepatosteatosis

**DOI:** 10.1038/s41598-020-73670-4

**Published:** 2020-10-09

**Authors:** Prabhakar Yellanur Konda, Vijayakumar Poondla, Krishna Kumar Jaiswal, Sreenivasulu Dasari, Reddemma Uyyala, Venkata Prasad Surtineni, Janardhan Yadav Egi, Anthony Johnson Antony Masilamani, Lakshmi Bestha, Sreenath Konanki, Muthukumaran Muthulingam, Lakshman Kumar Lingamgunta, Bindu Prasuna Aloor, Sridevi Tirumalaraju, Ankanna Sade, Venkata Ratnam Kamsala, Sreeharsha Nagaraja, Ranjani Ramakrishnan, Vijayakumar Natesan

**Affiliations:** 1 Department of Biochemistry, Krijan Biotech, Malleshwaram, Bangalore, India; 2 Department of Chemistry, Indian Institute of Technology Hyderabad, Sangareddy, India; 3 Department of Chemistry, Uttaranchal University, Dehradun, India; 4 Department of Biochemistry, Sri Venkateswara University, Tirupati, India; 5 Department of Organic Chemistry, Sri Padmavati Mahila Visvavidyalayam, Tirupati, India; 6 Department of Biotechnology, Sri Venkateswara University, Tirupati, India; 7 Department of Botany, Sri Krishnadevaraya University, Anantapur, India; 8 Department of Zoology, Sri Krishnadevaraya University, Anantapur, India; 9 Department of Biotechnology, Sri Krishnadevaraya University, Anantapur, India; 10 Department of Ecology and Environmental Sciences, Pondicherry University, Puducherry, India; 11 Department of Botany, Rayalaseema University, Kurnool, India; 12 Mahatma Gandhi National Institute of Research and Social Action, Banjara Hills, Hyderabad, India; 13 Department of Botany, Sri Venkateswara University, Tirupati, India; 14 Department of Pharmaceutical Sciences, College of Clinical Pharmacy, King Faisal University, Al-Ahsa, Saudi Arabia; 15 Department of Virology, Sri Venkateswara University, Tirupati, India; 16 Department of Biochemistry and Biotechnology, Annamalai University, Annamalainagar, Chidambaram, India

**Keywords:** Biochemistry, Biological techniques, Biotechnology, Microbiology

## Abstract

The high fat diet alters intestinal microbiota due to increased intestinal permeability and susceptibility to microbial antigens leads to metabolic endotoxemia. But probiotic juices reported for various health benefits. In this background we hypothesized that pectinase treated probiotic banana juice has diverse effects on HFD induced obesity and non-alcoholic steatohepatitis. 20 weeks fed HFD successfully induced obesity and its associated complications in experimental rats. The supplementation of probiotic banana juice for 5 months at a dose of 5 mL/kg bw/day resulted significant decrease (*p* < 0.05) in body weight (380 ± 0.34), total fat (72 ± 0.8), fat percentage (17 ± 0.07) and fat free mass (165 ± 0.02). Reduction (*p* < 0.05) in insulin resistance (5.20 ± 0.03), lipid profile (TC 120 ± 0.05; TG 160 ± 0.24; HDL 38 ± 0.03), liver lipid peroxidation (0.7 ± 0.01), hepatic enzyme markers (AST 82 ± 0.06; ALT 78 ± 0.34; ALP 42 ± 0.22), and hepatic steatosis by increasing liver antioxidant potential (CAT 1.4 ± 0.30; GSH 1.04 ± 0.04; SOD 0.82 ± 0.22) with normal hepatic triglycerides (15 ± 0.02) and glycogen (0.022 ± 0.15) contents and also showed normal liver size, less accumulation of lipid droplets with only a few congestion. It is concluded that the increased intestinal *S. cerevisiae* yeast can switch anti-obesity, antidiabetic, antioxidative stress, antioxidant and anti-hepatosteatosis effect. This study results will have significant implications for treatment of NAFLD.

## Introduction

Nutritionally probiotic foods are non pathogenic live microorganisms with high nutritive values, when consume adequately, confer health benefits to the host in various physiological functions; especially contribute to the health of gut microbiota^1^. Right now obesity or overweight is the most frequently encountered metabolic disease among the adults. High energy intake and less expenditure results obesity which shows impact on several metabolic and chronic disorders which includes insulin resistance, type 2 diabetes, hyperinsulinemia, hyperglycemia, hypertriglyceridemia, cardiovascular disease, hypertension, dyslipidemia, cancer, obstructive sleep apnoea, hyperuricemia, arthritis, and depression^2^. In obesity pro-inflammatory chemicals which released from the accumulated abdominal fat and make the body less sensitive to the insulin and disrupt the function of insulin responsive cells, known as insulin resistance which leads to oxidative stress, often linked to the development of T2D^3^.

In later stages, the oxidative stress induced by obesity involves in the pathogenesis of insulin resistance and accumulation of triglycerides (TG) in cytoplasm of the hepatocytes leads to pathogenesis of hepatic steatosis (fatty liver) without consumption of alcohol leads to non alcoholic fatty liver disease (NAFLD) or non-alcoholic steatohepatitis (NASH) which leads to fibrosis with an evolutionary course in cirrhosis and hepatocellular carcinoma (HCC)^4^. NAFLD is characterized by excess triglyceride (TG) deposition in the hepatocytes which mainly observed in subjects who fed on high-fat diets (HFDs) and significantly allied with a greater risk of insulin resistance, type 2 diabetes mellitus (T2DM) and cardiovascular diseases (CVD) and finally leads to liver dysfunction^5^. At present there is no pharmacological therapy for NAFLD or NASH. So, the management and treatment of NAFLD is a challenge. Recent experimental reports demonstrated that, the beneficial preventive effects of some non-pathogenic living microorganism strains like bacteria, yeasts on obesity by triggering immune responses not just in the gastrointestinal tract (GI) but throughout the body by mediating the stress responses^6,7^.

So, the probiotic food with natural non-pathogenic live microorganisms mediates stress responses generated by obesity and handle blood sugars better^8^. It is reported that intestinal microbial flora can influence health benefits by immunostimulation, improved digestion, absorption, inhibition of the growth of potential pathogens, cholesterol reduction, and vitamin synthesis^9^. The maintenance and controlling the microbial floral balance in the intestine achieved by supplementation of fermented probiotics is the best thought which affects lifestyle-related ailments^10^. Traditionally the banana fruit, *Musa paradisiaca* used for better digestion, diarrhoea, dysentery, intestinal lesions in ulcerative colitis, sprue, uremia, nephritis, gout, lower blood pressure, cardiac disease, stronger bones^11^. But, banana peels are considered as a waste, thrown without use. In fact, banana peels are nutritionally good source for fiber, magnesium, potassium, calcium, iron, vitamin B6, vitamin C, omega fatty acids, resistant starch, proteins, antioxidants, oxalates, phytates, saponins and also a good source of other types of fiber, such as pectin which is water-soluble. But, it is reported that pectinase enzymes are mostly produced from yeast cultures by using pectin as a substrate in sub-merged fermentation (SMF) techniques. Banana peel contains 35% weight of total banana fruit with a very much useful in production of valuable fermented products^12,13^.

Thus, in this study we investigated the effects of *Saccharomyces cerevisiae* probiotic yeast fermented from pectinase treated probiotic banana juice (PPBJ) on obesity induced by high fat diet (HFD) for 20 weeks which prone to develop hyperglycemia, hyperinsulinemia, hyperleptinemia, hypertriglyceridemia and hepatic steatosis. Finally, described the action of PPBJ in prevention and management of the obesity induced insulin resistance, type 2 diabetes (T2D), anti-oxidative stress with antioxidant activity and anti-hepatic steatosis effect by the modulation of intestinal microbiota with pectinase treated probiotic banana juice.

## Materials and methods

### Source of banana peels wastes

*Musa paradisiaca* (Robusta-AAA) banana variety was used for our experiment. The ripened banana peels wastes were collected from the local market and used within 24 h after collection. It was washed to remove the dust particles and shade dried in open air for 24 h later dried at 50 °C in a hot air oven until constant weight. The dry peels were milled for fine powders by using grinder and passed through 230 mesh (0.0024 inches, 63 microns) sieves to obtain banana peel powder and then the powder sterilized in UV irradiation for 1 h. The banana fruit peel powder (BFPP) was stored in clapped glass bottles and kept under refrigeration at 4 °C until use.

### Pectin extraction from banana peels and estimation

20 g of banana peels powder was weighed and mixed with double distilled water in 1:1.5 w/v ratios. The homogenate was acidified with lime juice and adjust the pH 2–2.5. The homogenate was autoclaved at 80 °C for 15 min. The mixture was cooled and filtered over cheese cloth, further filtration followed by Whatman filter of under vacuum. Then the filtrate was treated with isopropanol in the ratio of 1:1 to precipitate the pectin and kept on refrigerator for overnight. Subsequently, the mixture was allowed for centrifugation at 2000 rpm for 10 min. The residue was collected as pectin and dried at 48 °C for further use. The pectin content was determined using carbazole method^14^. Dried and ground banana peels were analyzed for moisture, non-reducing sugars, protein, cellulose, and lignin^15^. Reducing sugar concentration was estimated by using the dinitrosalicylic (DNS) acid method^16^. This mixture is then used as substrate or fermentation medium.

### Yeast strains

The starter pure culture of *Saccharomyces cerevisiae* yeast strains were isolated from fruit waste industries in lyophilized form by the method of Brooks^17^. The pure culture of yeast strains of *S. cerevisiae* grown at 37 °C for 24 h in YPD broth and was used as inoculum (3 × 106 cells/mL). Probiotic yeast strains were maintained separately in sterile YPD medium (Sigma) broth at 37 °C for 4 h, and then refrigerated at 4 °C. Subculturing was carried out for every 10 days.

### Inoculum size

The different inoculums size varies such as 2.5 ml, 5 ml, 7.5 ml and 10 ml of activated culture of *S. cerevisiae* was inoculated in a flask containing fermentation medium and pH 7.0 was adjusted by using 1 M NaOH. The strain was grown for 24 h in 250 mL Erlenmeyer flask containing 100 mL of liquid medium. Sufficient aeration was provided by agitation at 150 rpm at 30 °C.

### Production of pectinase enzyme by sub-merged fermentation (SMF)

The pectinase enzyme was produced by SMF method which was carried out in a medium composed of dried and ground banana peel (5% w*/*v), MgSO_4_ (0.03% w*/*v), (NH_4_)_2_SO_4_ (1% w*/*v), urea (0.15% w*/*v), FeSO_4_ (0.02% w*/*v), KH_2_PO_4_ (0.3% w*/*v), peptone (0.1% w*/*v), and distilled water (100 mL) in erlenmeyer flask (250 mL). The initial pH was maintained at pH 7.0 and flasks were sterilized at 121 °C for 15 min and inoculated with 2 × 10^7^ spores*/*mL. Then the cultured flasks were incubated at 30 °C for 5 d in a shaking condition (150 rpm) in rotary shaker. After incubation time period the samples were kept for filtration at regular intervals. The resultant culture filtrate was used as the enzyme source and stored at 4 °C for further analysis^18^.

### Enzyme assays for polygalacturonase (PGA) and pectin lyase (PL)

The activity of PGA was analysed by measuring the released reducing groups from the substrate (Banana peel). The reaction combination having 0.3 mL crude enzyme sample added to 1 mL of 1% of pectin substrate and 0.7 mL of 0.1 M acetate buffer (pH 4.5). The samples were incubated at 40 °C for 30 min. The reduced sugars were analysed through DNS procedure by Miller, 1959 using galacturonic acid as standard (Sigma, USA). One unit of PGA activity (U) was determined as the amount of the enzyme that liberates 1 μM of galacturonic acid per min.

The activity of pectin lyase (PL) was analyzed spectrophotometrically by measuring absorbance at 235 nm. The reaction solution contains 1 mL of 0.5% pectin (Himedia) dissolved in 0.1 M citrate phosphate buffer of pH 6.0, and 100 μL of crude enzyme was added to this substrate. The increase in absorbance was measured at 235 nm for 1–10 min at 25 °C. One unit of enzyme activity (U) was defined as the amount of enzyme which releases 1 μM of unsaturated uronide per min, based on molar extinction coefficient (5.55 × 103) of the unsaturated product^19^. Total soluble protein in the culture filtrate was estimated by the method of Lowry^20^ using BSA as a standard (Himedia, India).

### Effect of pH, temperature, and incubation time on enzyme production

The impact of the initial pH of the medium on enzyme production by *S. cerevisiae* in SMF method was investigated by altering the pH of the salt solution with 1.0 N HCl*/*1.0 N NaOH in a range of pH 3.0–9.0 in one-unit increments. The impact of temperature on enzyme production by *S. cerevisiae* in SMF method was investigated by incubating the inoculated flasks at different temperature conditions such as 20, 25, 30, 35, 40, and 45 °C. The influence of incubation time on enzyme production by *S. cerevisiae* in SMF method determined by incubating the inoculated flasks for 1–8 d. Finally, the enzyme activities were estimated for every 24 h.

### Scanning electron microscopy (SEM) analysis of banana peel

The banana peels of *Musa paradisiaca* (Robusta- AAA) were treated with an isolated crude enzyme to study the degradation of pectin present in the cell wall. The control and treated banana peels were examined under SEM. Before SEM analysis the fresh banana peels were washed, pulp was removed and peels were air dried. The dried banana peels were cut into small pieces of 10 × 20 mm in size and treated with 1 mL of isolated crude enzyme by incubating for 1 h but the control pieces were not treated with the enzyme. Both the control and treated samples were transferred into vials and fixed with 4% glutaraldehyde in 0.05 M phosphate buffer (pH 7.2) for 24 h at 4 °C. The samples were dehydrated in different series of graded alcohol and dried to a critical point with electron microscopy science CPD unit. Then dried samples were mounted over the stubs with double-sided conductivity tape. Finally, a thin layer of platinum was applied over the sample using an automated sputter coater (JEOL JFC-1600) for 90 s, and the samples were scanned under SEM (JEOL-JSM 5600) at various magnifications^21,22^.

### Effect of temperature on cloudiness of banana juice

*Musa paradisiaca* (Robusta- AAA) banana pulp was obtained from the fruit products. The banana juice was extracted by passing through a cheese cloth and for comparison 20 mL of banana fruit juice and 0.1% (w*/*v) each of crude and commercial pectinase solutions were added separately and incubated at various temperatures of 20 °C, 25, 30, 35, 40, 45, and 50 °C for 1 h (Bio-Tropicase, Biocon, Bangalore, India, specific activity 228 IU*/*mL). The mixture was heated on a boiling water bath and centrifuged at 805 × *g* and the transmittance (%T) of the supernatants of both enzyme treatments were measured at 650 nm^23^.

### Estimation of banana juice clarity

For 10 mL of banana juice, 0.1% (v*/*v) each of isolated and commercial enzymes (Bio-Tropicase) was added in two separate test tubes and kept for incubation at 35 °C for 60–300 min. Over the incubation time, the mixtures were centrifuged at 805 × *g* and the transmittance (%T) of the supernatants was measured at 650 nm. The total content of the pectin present in juice determined by estimating the anhydrogalacturonic acid of the solution using carbazole method^14^.

### Rat model

Albino wistar male rats weighing 180 ± 10 g were used and maintained at temperature (20–24 °C), 12 h light and 12 h dark cycle with relative air humidity 50%. The food and water provided timely throughout the experiment. Animal experiment was performed according to the animal ethical guidelines followed by the Institutional Animal Ethics Committee (IAEC).

### High fat diet composition (40% fat)

High fat diet (HFD) composition formulated as 40 g Fat (Beef thallow), 18 g Starch, 13 g Cellulose, 3.4 g Cysteine, 0.3 g Choline chloride, 22 g casein, 1.5 g mineral mix and 1.8 g vitamin mix. The HFD diet was prepared by using above ingredients available in the market.

### Animal experiment design

Albino Wistar rats male weighing 180 ± 10 g were randomly divided into 5 groups of 6 rats each; (n = 6).

Group 1: Control rats (C).

Group 2: Control rats treated with Pectinase treated Probiotic Banana Juice (C + PPBJ).

Group 3: High fat diet fed obese rats (HFD).

Group 4: High fat diet rats treated with pectinase treated probiotic banana juice (HFD + PPBJ).

Group 5: High fat diet fed rats treated with Orlistat (HFD + ORL).

Control rats (G-1) received a standard pellet diet alone. Control treated rats (G-2) orally supplemented with pectinase treated probiotic banana juice 5 mL/Kg bw (C + PPBJ). HFD obese control rats (G-3) received HFD alone (HFD). HFD fed obese rats (G-4) treated with pectinase treated probiotic banana Juice 5 ml/kg bw/day (HFD + PPBJ), and HFD fed obese rats (G-5) treated with Orlistat an anti-obesity standard drug 10 mg /Kg bw (HFD + ORL).

### Food intake, body weights and body composition

Food intakes of all groups of rats were recorded every day for 20 weeks. The body weights of all animals were recorded once in a week up to end of the study. Body composition for total fat, lean body mass, fat percentages and fat free mass were determined.

### Biochemical analysis

#### Measurement of glucose, glycosylated haemoglobin (HbA1c), leptin, and adiponectin

Fasting blood sugar estimated by glucose oxidase–peroxidase method^24^, Glycosylated hemoglobin (HbA1c)^25^, Leptin and adiponectin^26,27^.

### Insulin resistance (IR)

Plasma IR was determined by homeostasis model assessment. Homeostasis model assessment of insulin resistance (HOMA-IR) and β-cell function (HOMA-β) were calculated from fasting glucose and insulin values by using following formulae^28,29^.

$$ {\text{HOMA } - \text{ IR}} = \left[ {{\text{fasting}}\,{\text{glucose}}\left( {\text{mmol/L}} \right) \times {\text{fasting}}\,{\text{insulin}}\;\left( {\text{uIU/mL}} \right)} \right]{/22}.{5}; $$

$$ {\text{HOMA } - \text{ }}\beta = \left[ {{2}0 \times {\text{fasting}}\;{\text{insulin}}\left( {\text{uIU/mL}} \right)} \right]{/}\left[ {{\text{fasting}}\;{\text{glucose}}\;\left( {\text{mmol/L}} \right) - {3}.{5}} \right]. $$

### Oral glucose tolerance test (OGTT)

The OGTT was performed at the end of the experiment (end of 20 weeks) after overnight fasting animals. Glucose was administered (2 g/Kg bw) intraorally to all animals using a oral gavage needle. G-2 and G-4 rats administered with PPBJ and G-5 treated with Orlistat a standard antiobesity drug. Control rats (G-1) fed with distilled water alone. The blood samples collected from the tail veins of all groups of the rats from 0 to 120 min at every 30 min of the time interval. The blood sugar levels determined by using dextrostix with a basic one-touch accu-chek glucometer, OGTT performed according to the standard method^30^.

Finally, after 20 weeks of treatment, the rats were kept for overnight fasting. The blood was drawn from all the 5 groups’ animals and sacrificed by cervical dislocation. Blood was processed and plasma used for biochemical parameters. The organs like liver, kidney, muscle, heart, pancreas and adipose were collected and stored immediately at − 80 °C for further examinations. Among the tissue samples, liver tissue was used for histopathological analysis for Non-Alcoholic Steatohepatitis (NASH) determinations.

### Lipid metabolism

Plasma total cholesterol (TC)^31^. Triglycerides (TG)^32^ and high density lipoprotein cholesterol (HDL-C)^33^ were measured according to the manufacturer instructions of commercial kits.

### HFD induced obesity and liver damage

Obesity induced by feeding rats with 40% high fat diet for 20 weeks. HFD feed for long time causes oxidative stress which generates free radicals involved in lipid peroxidation leads to chronic hepatitis due to acute liver failure, hepatotoxicity and liver injury assessed by the determination of liver functional marker enzymes and characterized by the accumulation of lipid droplets within the hepatocytes which cause non-alcoholic fatty liver (hepatic steatosis).

### Liver marker enzymes

Liver injury was assessed by the determination of the serum enzymatic levels of Alanine transaminase (ALT), Aspartate transaminase (AST) were measured by enzymatic methods^34^. Alkaline phosphatase (ALP) was measured by P-Nitro Phenyl Phosphate method^35^.

### TBARS assay for oxidative stress in liver tissue (Lipid peroxidation)

Liver tissues homogenized with phosphate buffer pH 7.4 at 1:9 (w/v, tissue & buffer). The homogenates centrifuged at 2500 rpm for 10 min and the supernatants used for the subsequent determinations. The oxidative degradation of lipids in liver estimated by measuring the concentration of thiobarbituric acid reactive substances (TBARS) which express regarding malondialdehyde (MDA) content in tissues assessed as an index of lipid peroxidation^36^.

### Antioxidant enzymes activities in liver homogenates

The activities of Superoxide dismutase (SOD)^37^, Catalase (CAT)^38^, and Reduced glutathione (GSH)^39^ were measured in liver homogenates.

### Hepatic triglycerides and glycogen quantification

Liver TG’s extracted from a piece of 100 mg fresh liver mixed with a mixture of chloroform, methanol and dis. H_2_O in the ratio of 2:1:0.6 v/v in a high-speed homogeniser then centrifuged at 7000 × *g*, 15 min. The formed clot was mixed with a freshly prepared solution of chloroform-Triton (X100, 2%), evaporated at 55 °C and diluted with double distilled water. TG’s were estimated by using TG quantification kit according to the manufacturer’s guidelines (Abcam, Paris, France). Samples read at 550 nm and the concentrations were expressed in nmol/mg of liver. Liver glycogen content extraction was performed on 100 mg of fresh liver piece and expressed in mg of liver/mg of glycogen^40^.

### Histopathological analysis

The liver histopathological changes were assessed by hematoxylin and eosin staining (H&E). The liver specimens from all groups were fixed in 10% formalin solution and processed for paraffin embedding. 5 μm thick liver sections were produced and stained with H&E to evaluate the morphology and to verify changes of hepatic cells. Histopathological features of the slices were observed using a light microscope.

### Statistical analysis of biological data

Values were represented as mean ± SEM, and ‘n’ indicates the number of rats. The statistical significance compared to their respective controls ***p < 0.001; **p < 0.01; *p < *0.05*. Statistical comparison made using one way ANOVA.

### Ethical approval

All animal procedures were followed according to institutional ethics committee and were approved by the Animal Ethical Committee of Annamalai University.

## Results

### Pectin extraction from banana peels, substrate preparation, and yeast strains culture maintenance

*Musa paradisiaca* (Robusta- AAA) variety of banana peels were collected, dried, milled to fine powders and pectin was extracted. The content of pectin extracted from dried banana peel powder was 25%, w/w. The composition of dried banana peels shown in Table 1. The pectin extracted banana peels powder used as a substrate for pectinase enzyme production by using *S. Cerevisiae* sub culture in sub-merged fermentation (SMF) method.

**Table 1:** The composition of dried banana peel.

Constituents (%)*	Dried peel
Pectin	25.0 ± 0.07
Moisture	14.0 ± 0.02
Reducing sugars	30.0 ± 0.32
Non-reducing sugars	3.0 ± 0.26
Protein	6.0 ± 0.03
Cellulose and lignin	22.0 ± 0.15

*On dry weight basis (total solids = 80 ± 0.03).

Mean ± SE values (n = 3).

### SMF method for better production of polygalacturonase (PGA) and pectin lyase (PL)

*S. cerevisiae* was cultured by using the method of sub-merged fermentation (SMF) for the better production of PGA and PL for comparing the efficacy of enzyme production. Shade dried and ground banana peels were used as a substrate for fermentation. The enzymes production of both PGA and PL were higher in SMF. Higher production of pectinase is due to less catabolic repression.

### Effect of pH on the production of PGA and PL

**Figure 1:**
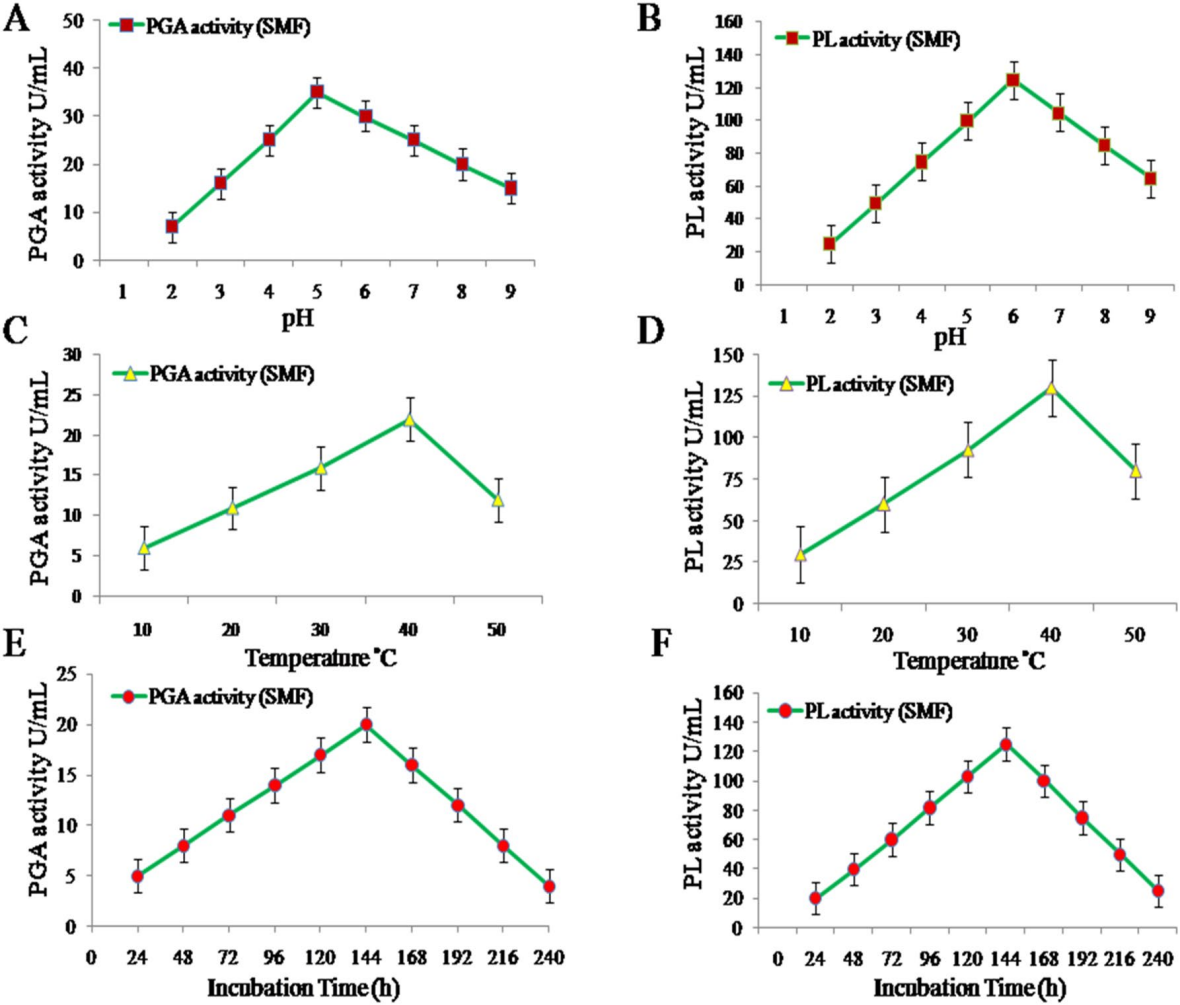
Effect of pH, temperature and incubation time on PGA and PL production in SMF method. (**A**) pH on PGA production. (**B**) pH on PL production. (**C**) Temperature on PGA production. (**D**) Temperature on PL production. (**E**) Incubation time on PGA production and. (**F**) Incubation time on PL production. *PGA* polygalacturonase; *PL* pectin lyase; *SMF* sub-merged fermentation. Data was presented as mean ± SEM (n = 3).

The initial pH in the medium varied from pH 3.0 to 9.0 for determining the effect of pH for the production of PGA and PL by *S. cerevisiae* in SMF. PGA production was more at pH 5.0 (Fig. 1A). There was low production of PGA activity at pH 3.0, and PGA activity declined further with an increase in pH 6.0 to 9.0. PL activity was more at pH 6.0 (Fig. 1B). PL activity was low at pH 4.0 and declined further with an increase in pH 7.0 to pH 9.0. In addition, the production of enzymes is also dependent on the pH of the medium. It was observed that pectinases produced have more stable properties, had broader pH profiles of enzyme activities.

### Effect of temperature on the production of PGA and PL

The effects of temperature on the production of PGA and PL by *S. cerevisiae* were carried out under different temperature conditions. The temperature conditions varied from 10 °C to 50 °C. The enzyme activity was low at 10 °C but the maximal activity was observed at 40 °C for both PGA (Fig. 1C) and PL (Fig. 1D). The enzyme activities were comparatively less at 10 °C and 50 °C. The lower temperature slows down the hydrolysis of pectin.

### Incubation time effect on the production of PGA and PL

The optimum incubation time required for the production of PGA and PL by *S. cerevisiae* is investigated. The Sub-Merged Fermentation (SMF) method was applied for 240 h, and the activities of enzymes were determined for every 24 h. In our study PGA achieved its maximum activity at 144 h (Fig. 1E). But, PL attained its maximal activity was at 120 h (Fig. 1F). There was very less activity was observed for both PGA and PL during first 24 h; after that the activity PGA was increased slowly and reached maximal at 144 h. Decrease in enzyme activity was observed before and after the optimum period for PGA. In comparison to this the activity of PL also increased after 48 h and reached maximum at 120 h. It decreased after the optimum period relatively slower than PGA. The decline in the PGA activity was observed after attaining the highest level of production, this could be due to the catabolic repression, cessation of the enzyme synthesis, and an increase of proteolysis in culture.

### Scanning electron microscopy (SEM) analysis of banana peel

**Figure 2:**
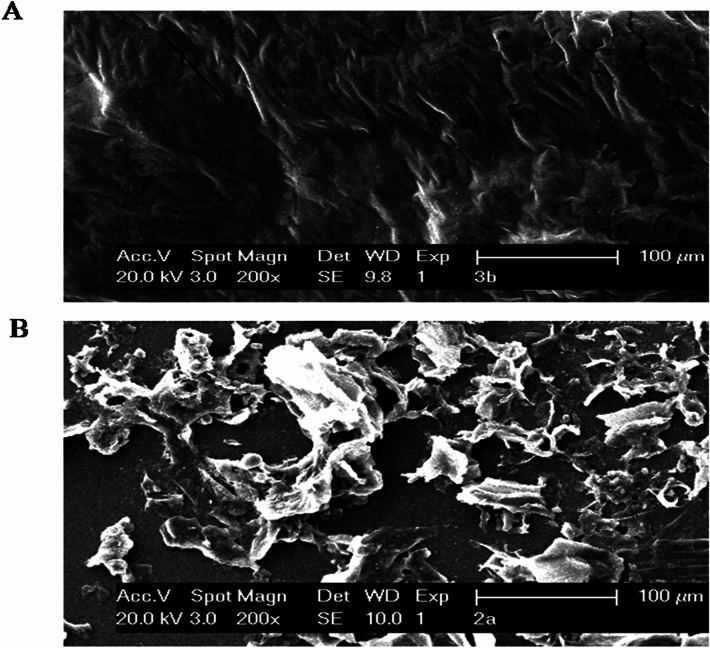
Scanning electron micrographs of *Musa paradisiaca* (Robusta- AAA) banana peel (**A**) enzyme treated and (**B**) control (scale bar = 100 μm).

Initially, the banana peels were treated with crude enzyme which produced from *S. cerevisiae* in SMF method to study the degradation of pectin present in the cell wall of banana peels. The banana peels of both control and treated were examined under scanning electron microscope but the banana peels treated with pectinase enzyme were softened by the degradation of pectin present in the cell wall. The SEM analysis revealed that the pectinase produced from *S. cerevisiae* caused greater pectin degradation, swelling up, separation of pulp microfibrils and pulp fibers (Fig. 2A) when compared to the control (Fig. 2B). In our study pectin lyases depolymerised the pectin more actively than pectate, and this was examined by scanning electron micrographs. From the analysis of SEM, we have concluded that pectin present in the cell wall of the banana peel was degraded when it was treated with the crude enzyme.

### Effect of temperature and incubation time on juice clarification by pectinase

The impact of temperature and incubation time on the action of pectinase enzyme produced from *S. cerevisiae* and commercial pectinase enzyme on banana juice were analysed. The maximum reduction in cloudiness was obtained at 35 °C incubated for 1 h. The temperature of 35 °C was found to be optimum for juice clarification for both isolated enzyme and commercial enzyme shown in Table 2. Banana juice was treated separately with the pectinase produced from the *S. Cerevisiae* and commercial pectinase for 5 h at 35 °C. The cloudiness of juice and the content of pectin were determined for every 60 min. The clear banana juice was observed when treated with pectinase produced from *S. cerevisiae*. There was a complete absence of anhydrogalacturonic acid observed at the incubation time of 60–180 min but appeared after 4 h (Table 3). Finally, we conclude that the banana juice treated with isolated pectinase enzyme provided maximum clarity compared with that of commercial pectinase enzyme.

**Table 2:** Temperature effect on clarification of banana juice by pectinase of *S. cerevisiae* and standard commercial pectinase.

Temperature (°C)	Pectinase (*S. cerevisiae*) (% T 650 nm) (blank 40%)	Commercial pectinase (% T 650 nm) (blank 40%)
10	25.2 ± 0.03	29.9 ± 0.17
15	39.1 ± 0.07	42.5 ± 0.21
20	46.7 ± 0.05	50.1 ± 0.08
25	58.0 ± 0.10	59.0 ± 0.02
30	69.8 ± 0.26	71.7 ± 0.34
35	80.2 ± 0.08	84.5 ± 0.02
40	72.0 ± 0.04	76.0 ± 0.06
45	65.5 ± 0.17	70.1 ± 0.12
50	58.8 ± 0.02	64.6 ± 0.03

Mean ± SE (n = 3); %T = % of transmission of light.

**Table 3:** Comparison of pectinase from *S. cerevisiae* and commercial pectinase in pectin degradation and clarification of banana juice at various incubation times at 35 °C.

Time (min)	Pectinase from *S.* cerevisiae clarity (%T)		Commercial pectinase clarity (%T)	
	(650 nm)	AGA* (%)	(650 nm)	AGA* (%)
0	60.4 ± 0.05	0	61.5 ± 0.01	0
60	69.0 ± 0.02	0	70.8 ± 0.04	0
120	77.8 ± 0.16	0	78.0 ± 0.15	0
180	84.5 ± 0.04	0	83.3 ± 0.27	1.02 ± 0.07
240	96.9 ± 0.12	10.0 ± 0.01	95.0 ± 0.64	1.40 ± 0.04
300	95.0 ± 0.07	10.5 ± 0.17	94.7 ± 0.55	1.65 ± 0.16

*AGA anhydrogalacturonic acid; mean ± SE (n = 3).

### Effect of PPBJ on control and HFD obese rats

#### Effect of PPBJ on food intake, body weight and body composition

**Figure 3:**
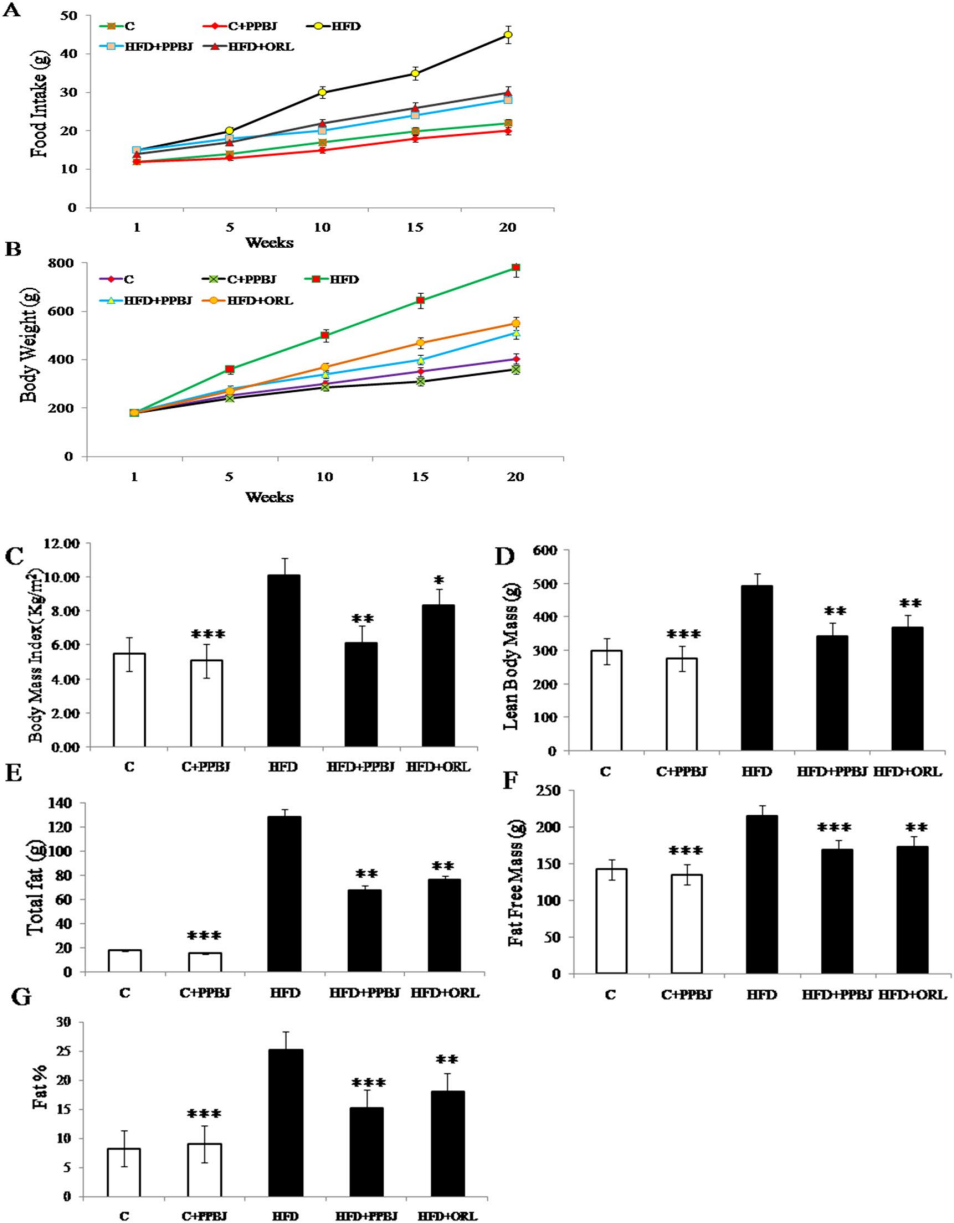
Effect of PPBJ on food intake, body weight and body composition in control and HFD induced obese rats for 20 weeks. (**A**) Food intake. (**B**) Body weights. (**C**) Body Mass Index. (**D**) Lean body mass. (**E**) Total fat. (**F**) Fat free mass and (**G**) Fat percentage. *PPBJ* pectinase treated probiotic banana juice; *HFD* high fat diet; *ORL* Orlistat. Data was presented as mean ± SEM (n = 6 each group). Statistically significance compared to their respective controls ***p < 0.001; **p < 0.01; *p < *0.05*. Groups compared using one way ANOVA.

The food intake, the changes in body weight and body composition of normal control and experimental group rats are shown in Fig. 3. Different food intake responses were noticed among the groups, an increase in the food consumption (40 ± 0.23) was observed in HFD group (G-3) when compared to the control group (18 ± 0.01) (G-1) (Fig. 3A). Although, HFD obese rats treated with probiotic pectinase treated banana juice (G-4) showed a marginal increase in food consumption (25 ± 0.04) (*p* < 0.05) when compared with control group (22 ± 0.01). After 5 weeks of diet, HFD induced an increase in body weight was observed and finally by the end of 20 weeks there was a significant increase in the body weight (700 ± 0.04) observed in HFD group (G-3) when compared to control (300 ± 0.012) (G-1). However, oral supplementation of PPBJ at a dose of 5 mL/kg bw/day significantly reduced the body weight of HFD treated obese rats (380 ± 0.34) (*p* < 0.05) (G-4) when compared to HFD control group (700 ± 0.04) (G-3) (Fig. 3B). A substantial increase in body weight indicates considerable changes in body composition. In this study BMI was significantly increased in HFD obese rats (10.10 ± 0.05) compare to control rats (5.5 ± 0.024) (Fig. 3C). Similarly lean body mass (493 ± 0.032) (Fig. 3D), total fat (130 ± 0.02) (Fig. 3E), fat free mass (220 ± 0.015) (Fig. 3F) and fat percentages (27 ± 0.42) (Fig. 3G) were found to be increased in HFD fed obese group when compared to control. However, oral supplementation of PPBJ significantly reduced the total fat (72 ± 0.8), fat free mass (165 ± 0.02), lean mass (330.4 ± 0.05) and fat percentage (17 ± 0.07) in HFD treated obese rats (G-4) (*p* < 0.05) when compared to HFD control group (G-3). Similar results were observed in rats fed with HFD + ORL (G-5).

### Effect of PPBJ on fasting glucose, HbA1c, insulin, leptin and adiponectin

**Figure 4:**
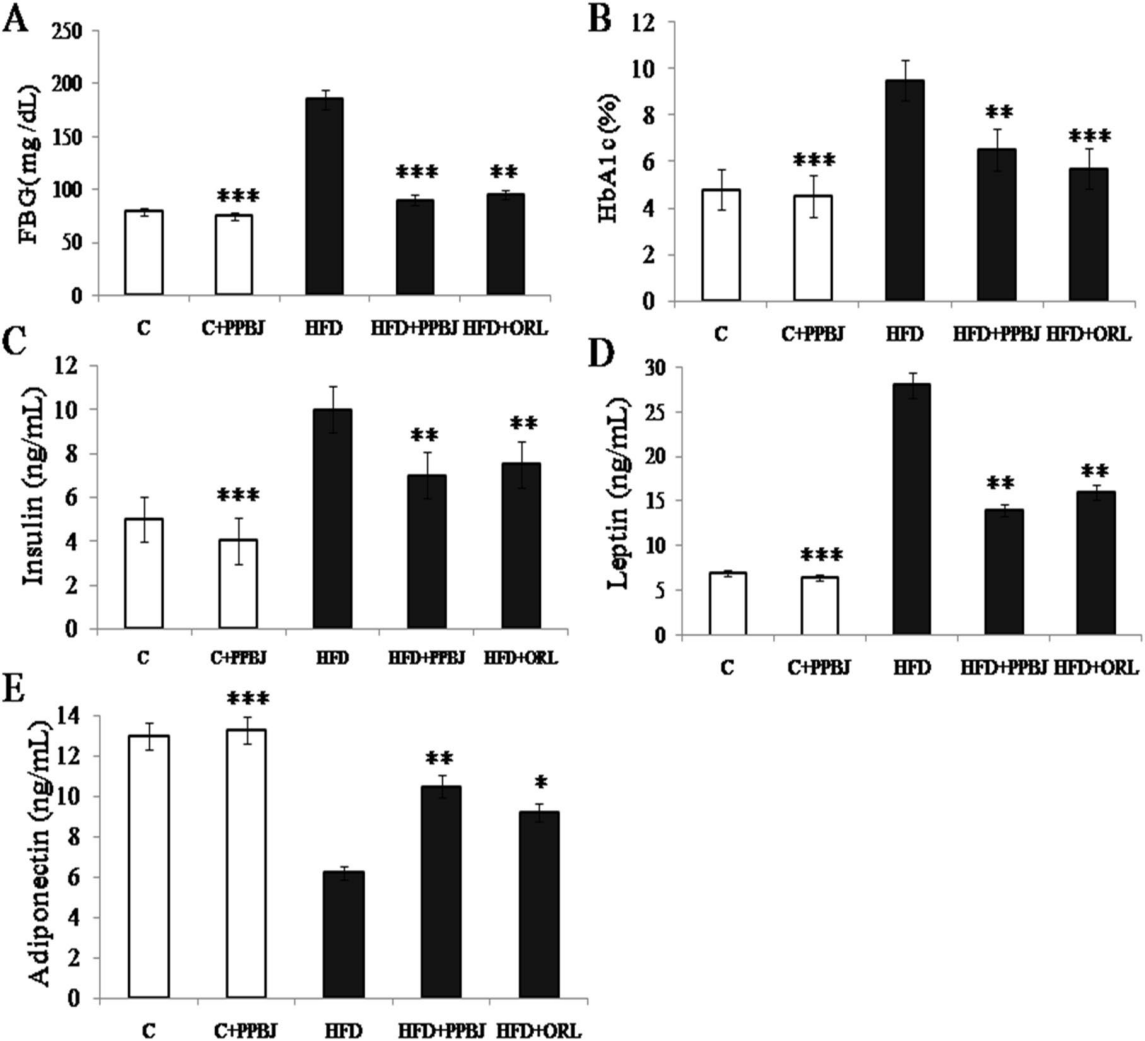
Effect of PPBJ on (**A**) Fasting blood glucose. (**B**) HbA1c (**C**) insulin, (**D**) Leptin and (**E**) Adiponectin. *FBG* fasting blood glucose; *HbA1c* glycosylated hemoglobin; *ORL* Orlistat. Data was presented as mean ± SEM (n = 6 each group). Statistically significance compared to their respective controls ***p < 0.001; **p < 0.01; *p < *0.05*. Groups compared using one way ANOVA.

After 20 weeks, in HFD fed obese control group there was a significant increase (190 ± 0.04) in fasting blood glucose was noted over control group (80 ± 0.01) (Fig. 4A). All the rats in HFD obese group maintained high fasting blood glucose until the end of 20 weeks. Oral supplementation of PPBJ tended to reverse the fasting blood glucose levels (100 ± 0.20) near to normal range (*p* < 0.05). HbA1_C_ levels of HFD obese group were significantly higher (9.8 ± 0.05) (G-3) than those in control group (4.7 ± 0.33) (G-1). Treatment with oral supplementation of PPBJ in HFD rats (G-4) reduced the levels of HbA1_C_ significantly (6.5 ± 0.11) (*p* < 0.05), indicating improvement in glycemic control (Fig. 4B). There was a significant elevation in insulin was observed in HFD obese rats (10.5 ± 0.43) (G-3) over control rats (5 ± 0.05) (G-1). Oral supplementation of PPBJ tended to reverse the insulin levels (7.2 ± 0.01) (G-4) at a dose of 5 mL/kg bw/day (Fig. 4C) (*p* < 0.05). Similar results were observed in rats fed with HFD + ORL (G-5).

Plasma leptin and adiponectin levels of both control and experimental obese rats are noted. In HFD fed obese group (G-3) a marked elevation in leptin level (28 ± 0.44) (Fig. 4D) and a decreased adiponectin level (6.2 ± 0.10) (Fig. 4E) were observed compared to control group (G-1). But, oral supplementation of PPBJ (5 mL/kg bw/day) has significantly decreased the leptin (13 ± 0.01) and increased the adiponectin levels (10.5 ± 0.05) in HFD treated obese rats (G-4) (*p* < 0.05). Similar results were observed in rats fed with HFD + ORL (G-5).

### Effect of PPBJ on insulin resistance

**Figure 5:**
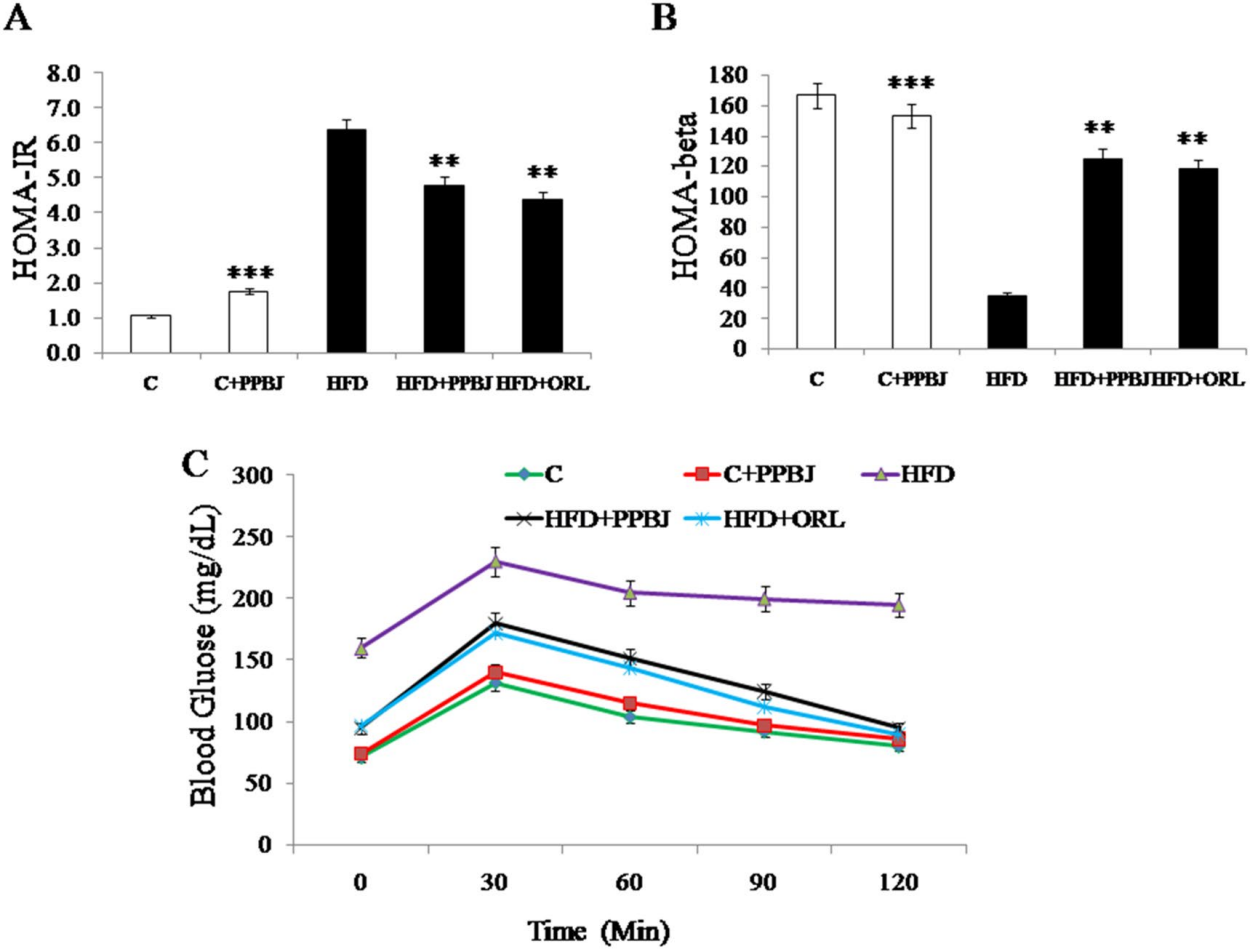
Effect of PPBJ on Insulin resistance. (**A**) HOMA-IR. (**B**) HOMA-beta and (**C**) OGTT. *HOMA-IR* homeostasis model assessment of insulin resistance; *HOMA-beta* homeostasis model assessment of β-cell function. *OGTT* oral glucose tolerance test; *ORL* Orlistat. Data was presented as Mean ± SEM (n = 6 each group). Statistically significance compared to their respective controls ***p < 0.001; **p < 0.01; *p < *0.05*. Groups compared using one way ANOVA.

The significant increase in the homeostasis model assessment of insulin resistance (HOMA-IR) was observed (6.6 ± 0.2) in HFD obese rats over control rats (1.0 ± 0.04) (Fig. 5A), confirming insulin resistance. But, the homeostasis model assessment of β-cell function (HOMA- β) level was significantly decreased (38 ± 0.11) in HFD obese rats (G-3) (Fig. 5B) when compared to the control rats (164 ± 0.21) (G-1). The oral supplementation of PPBJ in HFD fed rats (G-4) significantly decreased (5.2 ± 0.03) insulin resistance score and increased β-cell function (HOMA- β) level (125 ± 0.05) (*p* < 0.05). Similar results were observed in rats fed with HFD + ORL.

### Effect of PPBJ on OGTT

OGTT was performed to assess glucose tolerance, the results of the control and HFD fed obese rats are presented in Fig. 5C. In both control and obese rats maximum increase in FBG level was observed at 30 min after glucose load and declined to near to normal level at 120 min, But in obese rats, the increase in blood glucose level remained high over 120 min due increased insulin resistance leads to impaired glucose tolerance. The oral administration of PPBJ and orlistat in HFD obese rats resulted a significant decrease in blood glucose level at 60 min and beyond when compared with HFD obese control rats (*p* < 0.05).

### Effect of PPBJ on lipid metabolism

**Figure 6:**
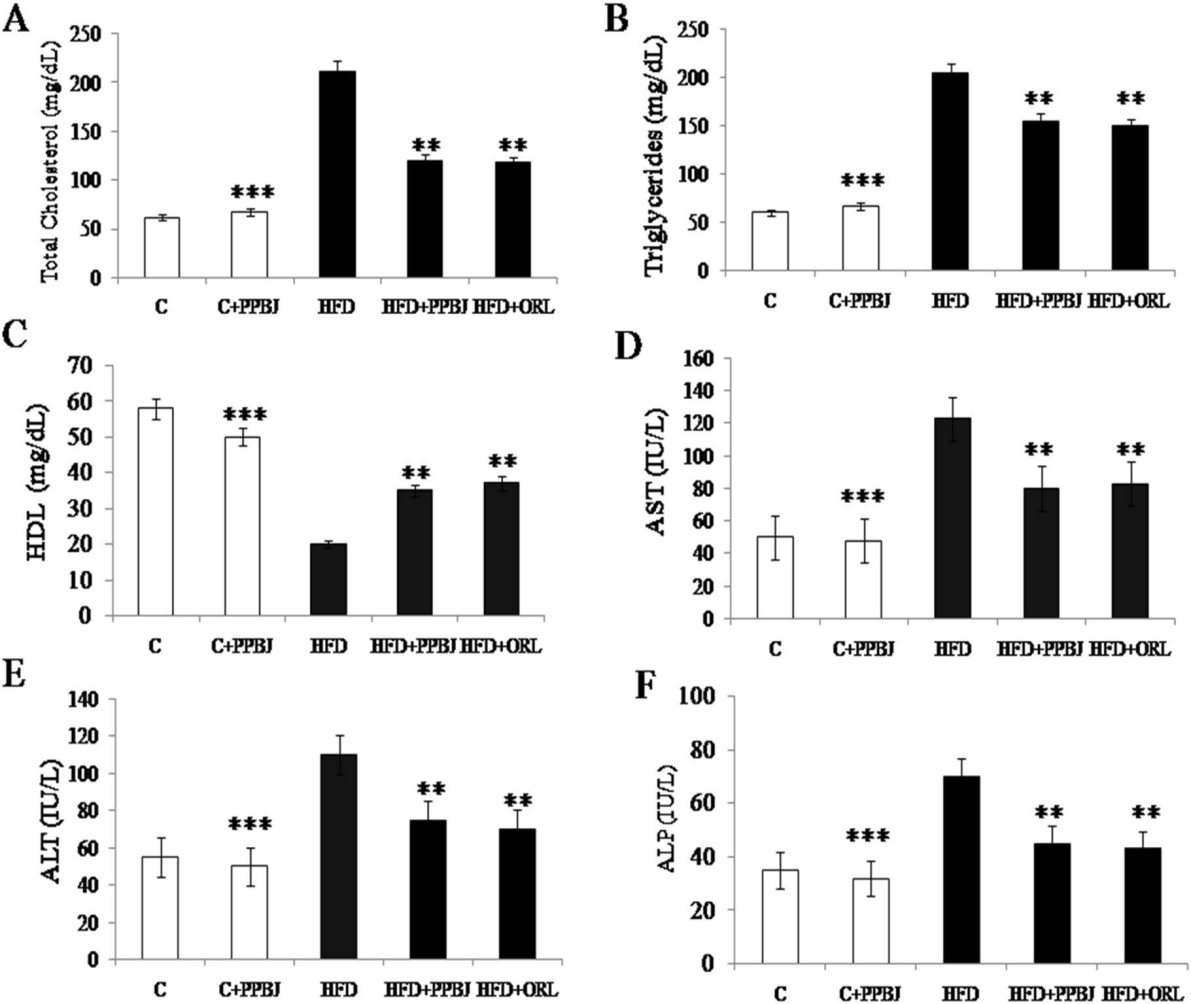
Effect of PPBJ on lipid metabolism and liver functional markers. (**A**) Total cholesterol. (**B**) Triglycerides. (**C**) HDL. (**D**) AST. (**E**) ALT and (**F**) ALP. *HDL* high density lipoproteins; *AST* aspartate transaminase; *ALT* alanine transaminase, and *ALP* alkaline phosphatase. Data was presented as mean ± SEM (n = 6 each group). Statistically significance compared to their respective controls ***p < 0.001; **p < 0.01; *p < *0.05*. Groups compared using one way ANOVA.

HFD fed obese rats (G-3) resulted in dyslipidemia changes with a significant marked elevation in plasma TC (210 ± 0.03) and TG (220 ± 0.22) and reduction in HDL levels (22 ± 0.14) as compared with control (G-1). However, oral supplementation of PPBJ for 20 weeks significantly reduced the levels of TC (120 ± 0.05) and TG (160 ± 0.24) with increased levels of HDL in HFD treated obese rats (38 ± 0.03) (G-4) (Fig. 6A–C) (*p* < 0.05). Similar results were observed in rats fed with HFD + ORL (G-5).

### Effect of PPBJ on liver functional markers

The levels of liver marker enzymes such as AST, ALT and ALP were evaluated as the markers of liver injury (Fig. 6D–F). HFD obese rats (G-3) showed considerably increased levels of plasma AST (124 ± 0.05), ALT (105 ± 0.12) and ALP (70 ± 0.25). But, oral supplementation of PPBJ (G-4) has decreased the levels of AST (82 ± 0.06), ALT (78 ± 0.34) and ALP (42 ± 0.22) respectively (*p* < 0.05). Similar results were observed in rats fed with HFD + ORL (G-5).

### In vivo hepatoprotective activity of PPBJ

HFD fed for long term induces chronic hepatitis, which increases fat accumulation in liver and subsequent oxidative stress causes cellular injury and inflammation. HFD feed for 20 weeks resulted fatty changes to the liver, which characterized by the accumulation of lipid droplets within the hepatocytes causes non alcoholic fatty liver (hepatic steatosis). HFD caused hepatic steatosis indicated by an increase in the weight of the liver which may be associated with an inflammatory process induced by the hepatic toxicity (G-3). But the rats treated with PPBJ (5 mL/kg bw/day) used to evaluate hepatoprotective capacity; the relative liver weights of the rats supplemented with PPBJ (G-4) were close to the control group (G-1). An increase of the liver weight may be associated with an inflammatory process induced by the hepatic toxicity (Table 4).

**Table 4:** Liver organ weights of different groups.

Group	Livers weight (g)
G-1	10.60 ± 0.22^a^
G-2	10.05 ± 0.03^a^
G-3	20.12 ± 0.37^b^
G-4	12.45 ± 0.08^a^
G-5	12.23 ± 0.45^a^

### Effect of PPBJ on liver lipid peroxidation (oxidative stress)

**Figure 7:**
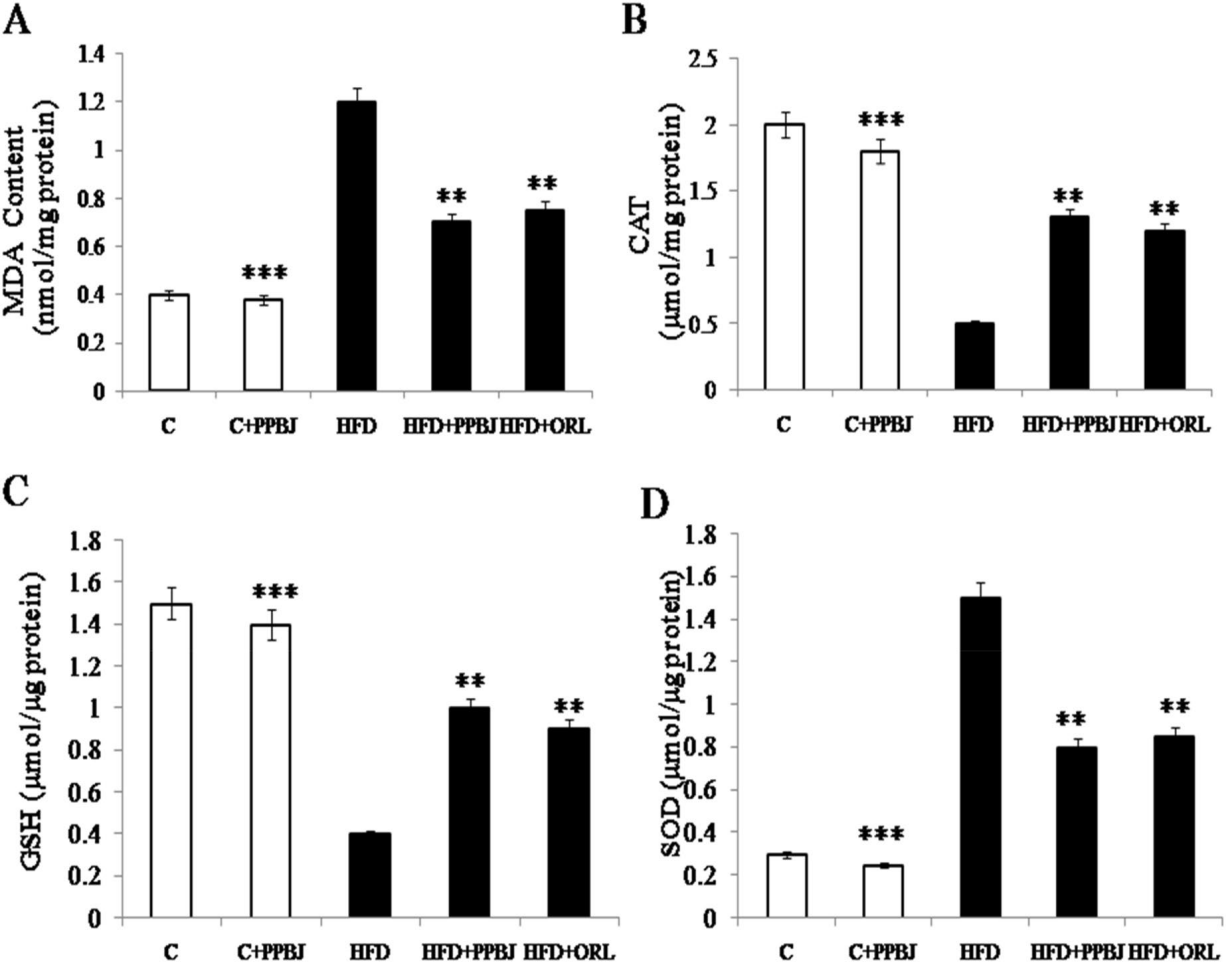
Effect of PPBJ on liver Lipid peroxidation. (**A**) MDA content. (**B**) CAT. (**C**) GSH and (**D**) SOD. *MDA* malondialdehyde; *CAT* catalase; *GSH* reduced glutathione; *SOD* superoxide dismutase. Data was presented as mean ± SEM (n = 6 each group). Statistically significance compared to their respective controls ***p < 0.001; **p < 0.01; *p < *0.05*. Groups compared using one way ANOVA.

In order to evaluate PPBJ ability to prevent oxidative stress, the oxidative degradation of lipids in liver assessed by evaluating the concentration of thiobarbituric acid reactive substances (TBARS) this was expressed regarding malondialdehyde (MDA) content. The hepatic MDA levels were found to be significantly increased in HFD obese rats (1.25 ± 0.04) (G-3) in comparison to control rats (0.4 ± 0.21) (G-1), whereas oral supplementation of PPBJ (G-4) resulted a significant reduction in MDA level (0.7 ± 0.01) (Fig. 7A) (*p* < 0.05). These findings indicated that PPBJ had good antioxidative stress property.

### Effect of PPBJ on antioxidants status

HFD induced obesity causes oxidative stress which generates free radicals and involves in macromolecules peroxidation leads to an MDA increase and a failure in the hepatocyte antioxidant system. Our investigation resulted a decrease in antioxidant defence with a significant reduction in the activities of CAT (0.5 ± 0.42) and GSH (0.4 ± 0.21) antioxidant enzymes and an increase in SOD activity (1.5 ± 0.55) in the livers of rats fed on the HFD (G-3) when compared with the control group (G-1) (Fig. 7B–D). This is due to increased lipid peroxidation lead to inactivation of the enzymes by cross linking with MDA; this will cause an increased accumulation of superoxide, H_2_O_2_ and hydroxyl radicals which could further stimulate lipid peroxidation during development of obesity. But the treatment with oral administration of PPBJ significantly elevated the activities of CAT (1.4 ± 0.30) and GSH (1.04 ± 0.04) but decreased the activity of SOD (0.82 ± 0.22) (G-4) (*p* < 0.05). Similar effects noticed with ORL with a fewer magnitude in comparison to those of the PPBJ.

### Effect of PPBJ on liver triglycerides and glycogen

**Figure 8:**
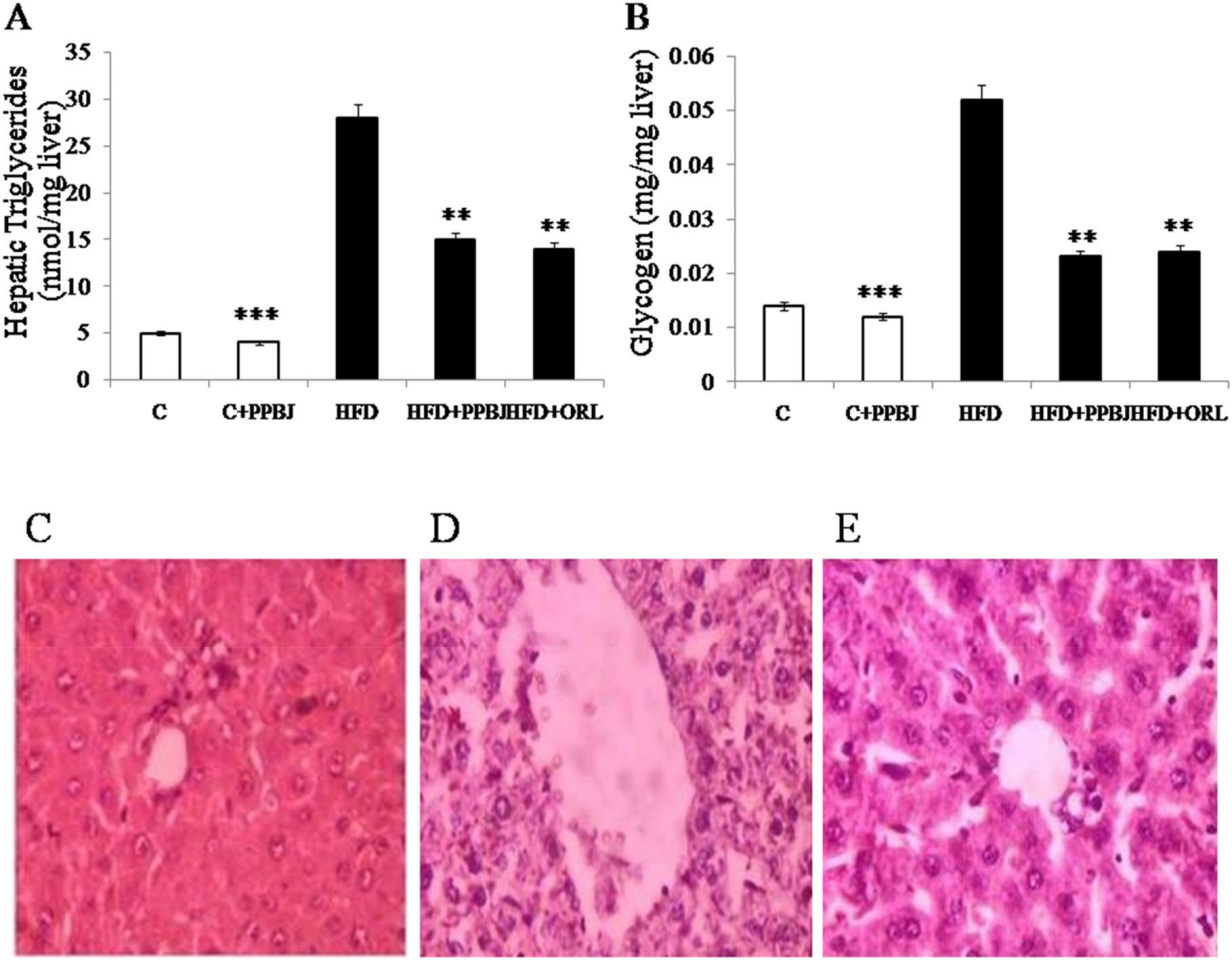
Effect of PPBJ on quantification of hepatic triglycerides, glycogen and, H & E staining for hepato steatosis. (**A**) Quantification of hepatic triglycerides. (**B**) Quantification of hepatic glycogen. (**C**) Control liver. (**D**) HFD obese liver (hepato steatosis) and (**E**) HFD treated with PPBJ liver. *PPBJ* pectinase treated probiotic banana juice; *HFD* high fat diet; *ORL* Orlistat. *H & E* hematoxylin and eosin staining. Data was presented as mean ± SEM (n = 6 each group). Statistically significance compared to their respective controls ***p < 0.001; **p < 0.01; *p < *0.05*. Groups compared using one way ANOVA.

Quantification of liver triglycerides (29 ± 0.01) and glycogen (0.052 ± 0.31) in frozen-liver tissues were found to be increased in HFD obese rats (G-3) compare to control rats (G-1) (Fig. 8A,B). Biochemical analysis of TG levels also confirmed the accumulation of lipids with a significant increase in triglycerides after 20 weeks. But oral supplement of PPBJ for 20 weeks (G-4) significantly reduced the levels of liver triglycerides (15 ± 0.02) and glycogen (0.022 ± 0.15) contents as in control (G-1) (*p* < 0.05).

### Histopathology of liver

Hematoxylin & Eosin analysis of the liver showed normal liver architecture in control rats (Fig. 8C) but HFD induced obese rats showed marked vacuolar degeneration and lipids accumulation on liver which indicated by increased liver size, larger lipid droplets, congestion with sinusoidal dilation, mild degree of lymphocytic infiltrate and bleeding (Fig. 8D). The rats fed with PPBJ group showed normal liver size with less the number of lipid droplets with only few congestion and mild lymphocytic infiltrate (Fig. 8E).

## Discussion

Banana fruits are most important part of human diet and frequently used for the production of juices, jelly recipe and jams due to its nutritional and medicinal values. But the banana peels were considered as wastes which bring about environmental pollution. The banana peel wastes could be harnessed into pectin, biogas and dietary fibres^41–43^. Industrially extracted pectin was used as a substrate for the production of pectinase enzyme by both solid state fermentation (SSF) and submerged fermentation (SMF) methods^44^. In this study, pectinase is sourced from *Saccharomyces cerevisiae* and produced by SMF. The impact of temperature and incubation time on the action of pectinase enzyme produced from *S. cerevisiae* and commercial pectinase enzyme on banana juice also analysed. Finally, it was concluded that the banana juice treated with isolated pectinase enzyme provided maximum clarity compared with that of commercial pectinase enzyme.

Excessive food intake with a high calorie is a major contributor to obesity among people and another large contributor to obesity is the lack of physical activity and sedentary behaviour^45^. As of today modern food environments were completely filled with nutrient poor and energy dense foods which highly palatable and processed in the ways that make it difficult for the body to regulate intake and weight. Probiotics are non pathogenic live microorganisms with high nutritive values, reported for maintenance of a healthier gut microbiota and an effective therapeutic adjuvant for various ailments like obesity, diabetes, cancer and age related disorders^46^. But the mode of mechanisms for their effectiveness on obesity, diabetes and its related complications are still not known. HFD can change the concentration of intestinal microbiota which causes metabolic endotoxemia and insulin resistance^47^. The molecular mechanisms involved in HFD and change in the intestinal microbiota environment are not fully evaluated, but high energy diets increase fatty acid oxidation in the liver by lipid peroxidation and increases malondialdehyde (MDA) production which leads to oxidative stress. The ROS generated reduce mucus production in the intestinal epithelium and make changes in the microbial environment^48^. Oxidative stress is a disequilibrium condition which triggers crucial risks for many metabolic diseases, such as obesity, diabetes, dyslipidemia, cardiovascular disease and also involves in the pathogenesis of liver diseases^49^. NAFLD is characterized by excess deposition of triglycerides (TG) in the hepatocyte followed by development of inflammatory NASH and fibrogenic responses. Currently, probiotics have been described to inhibit obesity associated complications and to delay the onset of diabetes in different experimental models including diet-induced, chemical-induced and genetically mutated animals^50^.

This investigation undertaken to utilize banana peel as appropriate substrate for the formation of pectinase enzyme by SMF method and fermentation of pectinase treated banana juice was carried out by newly isolated strain of *S. Cerevisiae* and characterized the fermented banana juice for various biochemical and histopathological parameters related to obesity, insulin resistance, oxidative stress, antioxidant activity and high fat diet induced hepatic steatosis. Thermal conditions for the maximal production of PGA and PL were studied in *S. Cerevisiae*. Both the enzymes showed maximal activity at 40 °C in SMF. SEM study indicates that pectinase from *S. Cerevisiae* degraded pectin compared to that of control banana peel when it is treated with crude enzyme. The cloudiness of the juice and the pectin content were determined every 30 min. Banana juice with good clarity observed when treated with pectinase from *S. Cerevisiae*.

In vivo evaluation of PPBJ significantly reduced the over weight of the body and liver organ weight in HFD induced obese rats and reduced high glucose levels by increasing insulin levels indicated by improved HOMA-IR implies a potential effect of PPBJ on glycemic control and significantly changed the gut microbiota composition with an increased proportion of *S. Cerevisiae.* Probioticated banana juice tested for lipid profiles, PPBJ significantly decreased the levels of TC and TG in HFD induced hyperlipidemia. Furthermore, HDL cholesterol levels were partly increased in hyperlipidemia. Liver is an elaborate system for antioxidant defences to eliminate or neutralize ROS. The generated free radicals cause lipid peroxidation and malondialdehyde (MDA) production in liver. The elevated levels of ROS influence the hepatocellular antioxidant defences, resulting in oxidative stress ultimately lead to hepatocyte injury, and liver cell death. In this study HFD supplementation attenuates HFD-induced hepatic steatosis which indicated by increased liver weight, increased intracellular enzymes levels, TBARS levels and accumulation of lipid droplets. After oral administration of PPBJ for 20 weeks treatment recovered the liver injury with normal liver weights, normal intracellular liver enzyme markers with decreased TBARS levels and MDA production by decreasing the oxidative stress.

The liver antioxidant enzyme activities CAT, SOD, and GSH also reached to normal level. These findings indicated that PPBJ had good anti-oxidative stress and antioxidant properties. The reports indicate that antioxidants can alter cholesterol absorption and increase antioxidant status. Therefore, PPBJ strongly prevented the failure of hepatocyte antioxidant system caused by obesity induced oxidative stress; thereby regulate the lipid and glucose homeostasis. Fermented PPBJ increase the gut microbiota and defends its host from pathogens by competitive exclusion, including occupation of attachment sites, consumption of nutrient sources, and production of antimicrobial substances. When the intestinal microbiota is abnormal, harmful bacteria will multiply excessively, inducing the endotoxin in blood and causing significant oxidative stress. Our investigation proves that probiotics alter the intestinal microbiota composition and inhibit the excessive proliferation of harmful bacteria.

Probiotics are live microorganisms which can live in the gastrointestinal tract that can regulate composition of the intestinal microbiome and also inhibit the uncontrolled growth of harmful bacteria, which may come up with decreased oxidative stress by influencing the production of β-defensin and IgA. The supplementation of probiotics could protect intestinal barrier by maintaining the tight junctions and inducing the production of mucin. The immunomodulation mediated by the probiotics may occur through the mediation of cytokine secretion by NFκB and MAPKs signaling pathways, also affects proliferation and differentiation of immune cells mainly T cells or epithelial cells. The probiotics can lower the intestinal pH and suppress the growth of pathogenic microorganisms by producing lactic acid, acetic acid, and propionic acid to maintain the proper balance of the gut microbiota^51,52^. The abnormal concentration of gut microbiome leads to the excessive proliferation of harmful bacteria which induces the release of bacterial endotoxins into the blood causes significant oxidative stress. But probiotic supplementation restore gut microbiota and protect the host from pathogens by competitive exclusion, including occupation of attachment sites, consumption of nutrient sources, and production of antimicrobial substances^53–55^.

Finally, it was clear that ad libitum high fat diet in rats leads to obesity which results structural and functional changes in the gut microbiota leads to dysbiosis and increased deposition of triglyceride in the adipose tissue. So that the gut microbiota has become a potential target to obtain optimal health. In this study, the primary mechanism of action of PPBJ is regulation of the host immune response and cytokine profile by the way of influencing the composition of the gut microbiome, protecting the host by displacing harmful bacteria, competing with pathogens for nutrients, and also by producing the antimicrobial substances such as lactic acid and acetic acid, creating a harmful microenvironment for pathogens by developing the immune system. Secondary, the modulation of gut microbiota by PPBJ treatment could affect body weight by increased energy expenditure, controlled consumption of food, and creating the satiety through gut peptide signaling, improved glucose tolerance, insulin sensitivity, lipolysis including decreased lipogenesis and adipogenesis could reduce the fat accumulation and reduced chronic systemic inflammation.

Histopathological studies also confirmed the liver damage caused by HFD in obese control rats presented degenerative changes of hepatocyte fat accumulation, inflammation and oxidative stress which contributed to cause hepatic inflammation leads to chronic liver injury. But treatment with PPBJ showed anti-inflammatory, anti-oxidative stress and antioxidant activities in rats and significantly suppressed the progression of chronic inflammation initiated by lipid accumulation within hepatocytes. So, PPBJ could be an effective therapeutic agent which suppresses the progression of fatty liver to NASH. Under these conditions, this antisteatotic effect of PPBJ is associated with the changes in metabolic reactions by increasing the microbiota, decreasing the oxidative stress and enhancement in the antioxidant potential of the liver.

## Conclusion

It is concluded that the increased intestinal *Saccharomyces cerevisiae* yeast can switch synergetic antioxidant effect and to an antagonistic effect towards the lipid radicals and may induce synthesis of polyunsaturated fatty acids which have anti-obesity, antidiabetic, antioxidative stress, antioxidant and anti-hepatosteatosis effect. The antisteatotic effect of PPBJ is associated with the enhancement in the antioxidant potential of the liver by reduction in the oxidative stress triggered by HFD. Banana juice increased the concentration of gut microbes and allowed the gut microbes to interact with the host’s liver tissues and to regulate its energy metabolism and also played a role in control of nutrient absorption and metabolism, the integrity of the gut barrier. This investigation is the evidence, suggesting the potential therapeutic action of pectinase treated probiotic banana juice on HFD induced obesity, obesity associated insulin resistance lipid peroxidation and hepatic steatosis prevention and treatment.

## Abbreviations

HFD: High fat diet
OGTT: Oral glucose tolerance test
NAFLD: Nonalcoholic fatty liver disease
T2DM: Type 2 diabetes mellitus
NASH: Non-alcoholic steatohepatitis
HCC: Hepatocellular carcinoma
CVD: Cardiovascular diseases
GI: Gastrointestinal tract
SSF: Solid state fermentation
SMF: Sub-merged fermentation
PPBJ: Pectinase treated probiotic banana juice
BFPP: Banana fruit peel powder
PGA: Polygalacturonase
PL: Pectin lyase
ORL: Orlistat
TOBEC: Total body electrical conductivity
OGTT: Oral glucose tolerance test
HbA1c: Glycosylated haemoglobin
HOMA-IR: Homeostasis model assessment of insulin resistance
ALT: Alanine transaminase
AST: Aspartate transaminase
ALP: Alkaline phosphatase
LPO: Lipid peroxidation
TBARS: Thiobarbituric acid reactive substances
MDA: Malondialdehyde
CAT: Catalase
SOD: Superoxide dismutase
GSH: Reduced glutathione
SEM: Scanning electron microscope
